# Food Security Among South Asian Americans: The Role of Availability, Affordability, and Quality of Culturally Appropriate Food

**DOI:** 10.3390/ijerph22081169

**Published:** 2025-07-24

**Authors:** Monideepa B. Becerra, Farhan Danish, Valentina Chawdhury

**Affiliations:** Department of Health Science and Human Ecology, Center for Health Equity, California State University, San Bernardino, CA 92407, USA

**Keywords:** South Asian Americans, food security, dietary practices, ethnic food availability, cost of food, quality of food, safety of food

## Abstract

**Background:** South Asian Americans (SAA) are one of the fastest-growing immigrant groups in the U.S. and face significant health disparities, particularly regarding chronic diseases like diabetes, hypertension, and cardiovascular disease. Dietary patterns play a crucial role in these disparities, with acculturation to Western diets linked to poorer health outcomes. Despite this, the impact of food insecurity on dietary habits among SAAs remains underexplored. This study aims to examine the availability, cost, and quality of ethnic food items and how food insecurity influences dietary practices in Southern California’s SAA population. **Methods:** The study was conducted in San Bernardino County, California, with field data collection focused on five South Asian ethnicity-specific grocery stores and three Western grocery stores. We assessed the availability and cost of key ingredients for commonly prepared SAA dishes. Additionally, focus group interviews were held with South Asian immigrants to understand food insecurity challenges and dietary adaptations. **Results:** The study found significant disparities in food availability and cost between SAA-ethnic grocery stores and Western stores. SAA stores were less accessible and more widely dispersed, with an average distance of 10 miles between them. While ingredients like ginger paste and cumin powder were available in both types of stores, items such as ghee, fenugreek seeds, and black gram were harder to find in Western stores. Focus group participants noted that ethnic foods, especially vegetarian ingredients, were more expensive than Western alternatives, leading many to substitute traditional meals with cheaper, less nutritious options. Participants also raised concerns about the poor quality of items in ethnic stores, such as expired produce, which further limited their food choices. **Conclusions:** Food insecurity, driven by limited availability, high cost, and poor quality of ethnic foods, poses significant challenges to the SAA community’s diet and health. Addressing these barriers could improve food security and health outcomes among SAA immigrants.

## 1. Background

South Asian Americans (SAA) are one of the fastest-growing Asian American groups in the United States (U.S.) with nearly 5.4 million residing in the country [1]. South Asians usually include those with national origins from Bangladeshi, Bhutanese, Indian, Maldivian, Nepalese, Pakistani, and Sri Lankan [2]. Further, the most recent U.S. Census data note that the Asian Indian alone population was the largest Asian alone group in 2020, with a 50% growth from 2010 to 2020 [3]. In recent years, empirical evidence has further highlighted health disproportions in this group, particularly concerning chronic and preventable diseases. For example, SAA have a higher prevalence of insulin resistance, type 2 diabetes, and early onset of type 2 diabetes [4,5,6] as well as obesity [7,8,9], when compared to other racial/ethnic groups.

Undoubtedly, addressing the factors associated with chronic disease burden is critical to ensuring the wellbeing of the SAA population. While some studies have addressed clinical factors, others have noted non-clinical, such as acculturation, physical activity, among others, with dietary practices being a major contributing factor [10,11,12]. For example, a study among 892 SAA found that consumption of fruits, vegetables, legumes, and nuts was associated with lower prevalence of metabolic syndrome and hypertension, while intake of animal protein, fried snacks, sweets, and high-fat dairy was associated with negative metabolic risk factors [13]. Acculturation studies have further explored that increasing generations of SAA in the U.S. was associated with past week fast food consumption at a rate 2.22 times higher when compared to first-generation counterparts [14]. Understanding the factors that lead to a shift in dietary practices, from traditional to more Westernized patterns, may offer insights into how to mitigate the dietary risk factors among this at-risk group.

The broader body of literature has highlighted the putative role of food insecurity in dietary practices, although there is limited research among SAA population. According to the United States Department of Agriculture households are considered food insecure if “*At times during the year, these households were uncertain of having or unable to acquire enough food to meet the needs of all their members because they had insufficient money or other resources for food*”, with 2023 date noting 13.5% households in the U.S. as food insecure. The empirical evidence highlights that food insecurity is related to worsening diet, higher rates of obesity, and overall negative health outcomes [15,16,17,18].

Studies among SAA, although limited, continue to highlight the burden of food insecurity. For example, a California-based population study noted that low acculturation was associated with the higher prevalence of food insecurity among SAA [19]. In a scoping review of food insecurity among Asian Americans, researchers also found prevalence food insecurity among SAA ranged from 3.14 to 38.2% [20]. Despite such evidence, there is a paucity of data on understanding food insecurity among SAA, and whether any key characteristics of food insecurity, such as availability, affordability, or quality influence shift dietary practices. Thus, the goal of our study is to address this gap. We hypothesize that SAA immigrants are likely to face food insecurity due to difficulties in finding or affording ethnicity-specific food items, which, in turn, may lead to the adoption of a more Westernized, high-fat diet.

## 2. Methods

### 2.1. Study Design and Data Collection

Geographic area: Our study was focused on San Bernardino County in Inland Southern California, due to its high rate of immigrant population, low socioeconomic status, and prevalence of food deserts. We employed a mix of field data collection followed by qualitative exploration of community perceptions.

Standards used: Our study design was informed in part by the USDA’s *Community Food Security Assessment Toolkit* [21], particularly the components focused on assessing community food resources and food store availability. We adapted elements of the Community Food Resource Inventory and Food Store Survey to explore the availability and affordability of culturally relevant food items specific to the SAA population in San Bernardino County. While the USDA toolkit emphasizes general food access and price comparisons, our study tailored this approach to ethnic food accessibility, a critical yet often overlooked dimension of food security in immigrant communities.

Specifically, we identified SAA-specific grocery stores and conducted systematic comparisons across three Western grocery stores stratified by price tier. Using a randomized selection of community-validated food and beverage items, we collected field data on both availability and cost, with supplemental input from store owners to ensure data accuracy. This localized, culturally tailored adaptation of USDA assessment methods enabled a nuanced understanding of how ethnic food accessibility intersects with structural barriers like socioeconomic status and geographic food deserts.

Field data collection: The first part of the study consisted of field data collection, during which we assessed the availability of SAA-ethnicity specific food items and cost of such items. First, we focused on identifying the availability of SAA-ethnicity-specific grocery stores in the county. Restaurants and eateries were not included in this search and emphasis was given to only grocery stores where the population would likely go to purchase ingredients for cooking food.

For comparative analysis, three Western grocery stores were selected, each representing a price income: Tier 1 was low cost, tier 2 was medium cost, and tier 3 was high cost. The tiers were determined by the description of each store, market value, and price range noted for each of the stores.

Next, we identified food items commonly consumed by the SAA population along with common ingredients used in the preparation of such food, with the exclusion of common condiments such as salt and pepper. During this phase, five random chosen food items (using random number generator) and one randomly chosen (same procedure) drink item was selected from a comprehensive list of community-reviewed food items. Next, a team of researchers visited each grocery store and collected the availability and cost of the ingredients. This data was collected with due consultation of the store owners and ensuring recent stocking delivery had occurred for items.

Qualitative data collection: The second part of the study consisted of qualitative data collection from community members, utilizing focus groups. Participants were recruited based on the following inclusion criteria: at least 18 years of age, immigrant to U.S., and self-identification of South Asian ancestry. Face-to-face focus groups were conducted using two central questions and additional probe questions. The study was approved by the institutional review board and all participants were asked for informed consent. Only those provided such consent were asked survey questions and their data was utilized in this study.

### 2.2. Data Analysis

Using the standard mapping software, SAA-specific ethnic grocery stores were identified based on name and description, including phone calls if needed. Next, we created a table to list each of the common food items along with the ingredients needed to cook them. To ensure cultural competency, experts of South Asian ethnicity were consulted to identify such items. After each of the identified store was visited, the price and availability of each ingredient was logged and calculated. Finally, a comparative analysis was performed to assess the availability and cost of ingredients by store type.

When analyzing ingredients, several considerations were made. Various meats are used in the preparation of *curry* (region-specific), in addition to some geographic areas only using vegetables due to vegetarian diet. As such, only the ingredients needed to cook a standard *curry* were used in this study. Likewise, *sabzi* is a general term for a vegetable dish where each individual can cater the dish to their liking of vegetables. As such, we only selected the ingredients needed to create the base, as individuals can change the vegetables based on availability. On the other hand, lack of the basics needed would prevent cooking of the dish. In addition, for each of the food items enlisted, there were a variety of brands available. The most common brands purchased, as noted by discussions with store owners, were selected in the research. For example, the most common brand for basmati rice was “Royal” and for *ghee*, the most common brand purchased was “Gopi.” Similarly, some recipes called for rice while others noted basmati rice. For the purposes of this analysis, the availability of any type of rice was logged.

The frequency at which each ingredient was found was entered into Microsoft Excel and then exported to SPSS (version 27) for analysis. The cost of each ingredient and each store was collected, and SPSS was used to find mean cost among SAA-ethnic specific stores and Western grocery stores for comparison purposes. Given that different stores had prices by varying degrees of weight measurement, all data were converted to ounces in order to compare across stores. Descriptive and bivariate analyses were conducted to assess frequency of availability, average cost, as well as differences between types of stores.

Qualitative responses from focus-group interviews were transcribed verbatim. Next, thematic analysis was conducted by identifying common words/phrases and then grouping them into common categories and finally giving rise to cumulative themes. This process continued until we reached theoretical saturation where no new themes emerged.

## 3. Results

### 3.1. Availability

We identified a total of five grocery stores in SBC. The identified SAA-ethnicity specific stores were spread at random and the distance from one to another was found to be at an average of 10 miles. Each city evaluated had an average of one South Asian store within its area. On the other hand, Western grocery stores are seen to be in abundance and are in closer proximity to each other. Next, as shown in Table 1, of the six common food/drink items for SAA ethnicity, 17 ingredients were identified that were needed to make food/drink, many of which were repetitive. We then evaluated the availability of all 17 ingredients per store.

As shown in Table 2, the availability of such ingredients was varied across stores, with only one having all the ingredients needed. For example, in order to create the popular dish *daal* (lentils), the ingredients needed are orange lentils, ghee, garam masala, ginger paste, garlic paste, and cumin powder. Both orange lentils and *ghee* were found in 60% of SAA-ethnic specific stores, but in 100% of the Western Stores. On the other hand, garam masala was available in 80% of SAA-ethnic specific stores and 33% of Western stores. Ginger paste, garlic paste, and cumin/cumin powder were available in 100% of SAA-ethnic specific stores and Western grocery stores. Some of the ingredients for making *sabzi* (ginger paste, garlic paste, and cumin/cumin powder) were found in 100% of SAA-ethnic specific stores and Western grocery stores. On the other hand, fenugreek seeds were only available in 60% of SAA-ethnic specific stores and 33% of Western grocery stores. Additional food item-specific analyses are included in Table 2.

Focus group participants also noted that the availability of ethnic-specific food ingredients was limited and/or would require significant travel to obtain them.

*“Sometimes that we can’t find the proper ingredients that we usually used to cook in our country, but we’ll have to be accustomed to the ingredients we get here.”*

*“I don’t think South Asian people living in Inland Empire are able to find their ethnic group food.”*

*“And most times when people go far like L.A, they buy some stuffs which are not much available around here.”*

*“It depends very strongly on where you live.”*

*“…the stores where I can find these ethnicity specific food items, they’re far from our house. So, we have transportation problems and many other problems to go. So, it’s hard to find.”*

### 3.2. Quality

A unique theme that emerged specifically when discussing South Asian grocery stores was that of safety and quality of food items. Participants often noted poor quality of ingredients that were found, either in comparison to expectations or what they were used to in their home country, with words such as “*bad quality*” or “*expired*” used to refer to the food items. The researchers did not assess expiration dates during the time of the field data collection as that was not part of our approved protocol. The issue of quality of food was also a novel theme that emerged from the focus groups.

### 3.3. Affordability

As shown in Table 3, culturally specific ingredients were either missing entirely from mainstream grocery stores like Walmart and Food 4 Less, or when available, typically at Whole Foods, priced substantially higher, with several items exceeding $7 per unit. For example, black gram, a staple for dishes like *dosa*, was not found in any of the three surveyed grocery chains. Key spices such as garam masala, cardamom, and fenugreek seeds were inconsistently available, often limited to only one store (typically Whole Foods), and sold at a premium price, with some items exceeding $7 for small quantities. Even basic components like ghee and lentils varied widely in availability and cost.

These findings were later echoed and contextualized by focus group participants, who described how such cost and availability constraints shaped their everyday food choices. Particularly, a participant highlighted that what is considered healthy in their home country would be expensive in the U.S. and thus switching to cheaper options in the new country was norm. Furthermore, it was not just limited to what was considered healthy, but even cultural/religious appropriate food items were limited due to cost and often impacted participants’ dietary behavior.

*“Definitely, because there’s a difference in the healthy food you cook in India as to the stuff you cook here. Healthy stuff like lettuce [are] nine cents. But in India if it’s a healthy food maybe you would make Dosa or Daliya, which is really expensive [here]. Dalia 1 lb is like 5.99 in Indian stores but lettuce I can get for 99 cents apiece. So, I switched to a healthy food which is non-Indian to save money.”*

*“I used to eat in India parathas for breakfast. Here, what I get an Indian store are frozen parathas and are not much healthy. So, I have to eat sandwiches.”*

*“One of the problems is that, because like I used to eat halal meat OK…halal meat is super expensive.”*

Furthermore, participants noted that ethnic-specific food items were more expensive, especially if they were vegetarian food items.

“*Well buying vegetarian food here is expensive because I guess buying non-vegetarian food at a restaurant or even buying raw meat is cheaper than buying vegetables and cooking them at home.”*

*“What I usually like to have, for example the fish and vegetables I like are really expensive to get.”*

*“… certain type of meats is quite cheap over here compared to the vegetables or certain group of meat or fishes.”*

## 4. Discussion

This study explored the availability, cost, and quality of South Asian ethnicity-specific food items in Inland Southern California. The findings indicate significant disparities between SAA-ethnic specific grocery stores and mainstream Western grocery stores, both in terms of availability and cost, which may contribute to changes in dietary practices and food security challenges for the SAA community.

### 4.1. Availability

The availability of SAA-specific food items was limited across the region, with a stark contrast between the proximity and availability of these items in SAA-ethnic specific stores and Western grocery stores. While there were five identified SAA-specific stores across the county, they were scattered and, on average, 10 miles apart. In contrast, Western grocery stores were abundant and clustered together, making access to mainstream food options significantly easier. The concentration of Western grocery stores in the area may reflect a broader trend of market consolidation, where chain stores dominate urban and suburban areas, leaving immigrant communities with fewer options for purchasing culturally familiar foods. While similar studies in the U.S. are lacking, an intervention in Canada highlighted the efficacy of incentivizing ethnic-grocery stores in alleviating food insecurity among low and mixed-income minority groups [22].

In terms of food item availability, only one SAA-ethnic specific store had all the ingredients necessary to prepare the popular dish *dal*, compared to 100% availability in Western stores. While some ingredients like ginger paste, garlic paste, and cumin powder were readily available in both store types, others, such as fenugreek seeds and ghee, were far more difficult to find in Western grocery stores. These findings align with other research that has noted the challenges immigrants face in accessing ethnic food ingredients, particularly in areas where the market is less attuned to their cultural needs. Qualitative data from focus groups of our study further emphasized this issue. Participants expressed frustration with the lack of ethnic-specific stores in their vicinity and the significant distances required to travel to obtain familiar ingredients. While similar studies in the U.S. are lacking, assessment in other Western nations with high immigrant populations have noted a similar trend [23]. For example, a cross-sectional assessment in Australia noted that immigrants were likely to travel longer distances to acquire culturally appropriate food as compared to when needing to acquire Western-based food [24].

Cumulatively, such results highlight that continued barriers to obtaining desirable food may exacerbate food insecurity, leading many to substitute culturally significant foods with more readily available and less nutritious alternatives.

### 4.2. Cost and Dietary Shifts

Cost emerged as a key theme when participants discussed their food choices. As highlighted by participants, the affordability of healthy foods, especially vegetables, in the U.S. is markedly different from their home countries. Items such as lettuce and rice are cheaper in the U.S., while traditionally prepared South Asian dishes, such as *dosa* and *dal*, are often expensive, making them less feasible on a tight budget. In our study, participants reported substituting their traditional foods for more affordable Western food. This pattern of dietary adaptation is consistent with the concept of “nutrition transition,” where due to economic pressures, immigrants often shift from traditional, nutrient-dense diets to cheaper, less healthy alternatives. While such studies for SAA lack in the U.S., the assessment of immigrant SA in Europe have noted a similar shifts towards high fat, high refined carbohydrates, and low fiber consumption, which may lead to poorer nutritional outcomes over time [25].

The cost of ethnic-specific foods, especially vegetarian options, was a significant concern for many participants. As noted, the price of vegetarian ingredients, such as fresh vegetables and rice, was often higher than non-vegetarian items, contributing to the preference for cheaper meat-based meals. This is in line with findings from previous studies in other nations where cost of food was a contributing factor in poor dietary practices among South Asian immigrants [26]. The higher cost of healthful, culturally appropriate foods in low-income areas may perpetuate cycles of food insecurity and poor dietary habits.

### 4.3. Food Quality and Safety Concerns

A unique theme that emerged in this study was participants’ concern over the quality and safety of food items purchased from ethnic-specific stores. Several participants remarked on the poor quality, or even expiration, of ingredients in these stores, which may contribute to mistrust in the availability of fresh, nutritious options. Concerns over food quality may further discourage individuals from purchasing from these stores, driving them to rely on mainstream supermarkets that offer more consistent quality standards, even if these options are culturally incongruent and in turn pushing immigrant populations to rely on cost-saving but unhealthy food items.

Cumulatively, the limited availability and high cost of ethnic-specific food items, combined with concerns about food quality, have significant implications for food security and health outcomes in the SAA community. As the results suggest, food insecurity is not only a result of the inability to access affordable food, but also of the cultural dissonance between the foods immigrants are accustomed to and what is available in mainstream grocery stores. The lack of access to culturally appropriate food items may drive individuals to adopt less healthy Western diets, contributing to rising rates of obesity, diabetes, and other chronic conditions.

### 4.4. Limitations and Strengths

While the study provides valuable insights into the availability, quality, and affordability of ethnic-specific foods, it has its limitations. First, the sample size of stores was relatively small (five SAA-specific stores and three Western stores), which may not fully capture the diversity of grocery store options across the state or the broader regional variations in food access.

Additionally, the focus groups, while valuable in exploring the perceptions of food insecurity, represent a limited sample of the SAA community, and further studies should seek to include a more diverse range of participants in different areas of the county or beyond quantitative means. We also did not assess detailed demographics of the participants due to small ample size and ease of identification, in an already small population.

Notwithstanding such limitations, the present adds to the body of literature in several ways. First, this study examines affordability, availability, and food safety in recognition that food security is defined not solely by access, but also by the consistent availability of safe, culturally appropriate, and economically attainable food. By addressing these dimensions together, we provide a more comprehensive assessment of food security within the SAA immigrant community. Importantly, this multidimensional approach aligns with the USDA’s definition of food security, which emphasizes not only having enough food, but also having food that meets nutritional and cultural needs in a safe and socially acceptable manner.

Further, our inclusion of field-collected cost data, geographic access mapping, and firsthand community narratives allows for a fuller understanding of how intersecting barriers, such as high costs of staple ingredients, poor quality of available items, and distance to ethnic-specific stores, contribute to chronic food insecurity. Moreover, by integrating affordability and safety alongside availability, this study moves beyond traditional food environment audits to provide a culturally responsive model that can be adapted for use in other immigrant communities facing similar challenges.

Finally, there remains a paucity of food security assessments focused on the SAA population, despite it being one of the fastest-growing immigrant groups in the U.S. [1]. The results of this study highlight the need to prioritize this vulnerable population through tailored population health interventions.

### 4.5. Implications for Practice

Findings of this study reveal clear implications for improving food access and nutritional equity among SAA immigrant communities as culturally specific food insecurity is not only a matter of physical access but also of affordability, cultural relevance, and quality, factors often overlooked in mainstream food security policy and intervention design [27,28].

For example, public health practitioners and urban planners should prioritize supporting ethnic grocery retailers in underserved areas. These stores often serve as vital community anchors by offering culturally familiar foods not available in chain supermarkets. Local governments and food policy councils could provide small business incentives, zoning support, or grant funding to help these retailers expand inventory, reduce costs, and maintain food quality. Consumer preference for grocery stores that offer healthy and diverse food options has been well documented in the literature [29]. Therefore, expanding the availability of culturally relevant foods may help improve the nutritional outcomes of the SAA population.

In addition, researchers and practitioners should collaborate to develop and validate community food security assessment tools that explicitly consider cultural dimensions of food access. While the USDA’s Community Food Security Assessment Toolkit provides a valuable foundation, it lacks specificity for evaluating access to ethnic-specific ingredients, affordability of cultural meals, and the psychosocial impact of dietary displacement. Studies show that culturally adapted food-environment tools, like the Latino NEMS-S [30], more accurately capture the unique offerings in ethnic grocery stores compared to standard versions. This supports the need for similar adaptations tailored to SAA and other immigrant communities. Together, these practices can move the field beyond generic “food access” models toward equity-driven, culturally grounded strategies that reflect the lived realities of immigrant communities.

## 5. Conclusions

This study highlights significant barriers to food access faced by the SAA immigrant community, with limited availability and high costs of ethnic-specific food items playing central roles in shaping dietary practices. The findings underscore the need for policy interventions aimed at improving food access in immigrant communities, addressing both cultural and economic barriers. Supporting ethnic grocery stores, reducing the cost of healthful, culturally appropriate foods, and improving the quality of available ingredients could mitigate food insecurity and promote better health outcomes for such a vulnerable group.

## Figures and Tables

**Table 1:** Five most commonly cooked SAA food and drink items.

Food Item	Common Ingredients Used
Dal (lentils)	Orange lentils (Masoor Dal), ghee, garam masala, ginger paste, garlic paste, cumin/cumin powder.
Sabzi (vegetable mix)	Ginger paste, garlic paste, cumin, fenugreek seeds, fennel seeds.
Dosa (rice pancake)	Rice, Black gram.
Pulao (rice dish)	Rice (usually basmati rice), bay leaf, cinnamon/cinnamon stick, dried onion.
Curry (base mix)	Ginger paste, garlic paste, garam masala, cinnamon, cloves, cardamom.
Chai (tea)	Milk, tea powder/leaf.

**Table 2:** Food/drink-item specific ingredient availability. X = found in store.

	South Asian American Store						Western Stores			
	Store 1	Store 2	Store 3	Store 4	Store 5	Total	Tier 1	Tier 2	Tier 3	Total
Orange lentils	X	X	X			60%	X	X	X	100%
Ghee	X	X	X			60%			X	33%
3Gar5am masala	X	X	X	X		80%			X	33%
Ginger paste	X	X	X	X	X	100%	X	X	X	100%
Garlic paste	X	X	X	X	X	100%	X	X	X	100%
Cumin powder	X	X	X	X	X	100%	X	X	X	100%
Fenugreek seeds	X			X	X	60%			X	33%
Fennel seeds	X			X	X	60%		X	X	67%
Rice	X	X		X		60%	X	X	X	100%
Black gram	X	X		X		60%				0%
Bay leaf	X	X			X	60%	X	X		67%
Cinnamon stick	X	X			X	60%	X	X	X	100%
Dried onion	X	X			X	60%				0%
Cloves	X		X	X	X	80%	X	X	X	100%
Cardamom	X		X	X	X	80%		X	X	67%
Tea powder	X	X	X			60%	X	X	X	100%

**Table 3:** Comparative cost and availability of South Asian food ingredients by store.

Dish	Food 4 Less Avg Cost (USD)	Walmart Avg Cost (USD)	Whole Foods Avg Cost (USD)	Missing Ingredients	Most Expensive Store	Fully Available?
Dal	17.25	16.24	25.44	Ghee, Garam Masala	Whole Foods	No
Sabzi	11.27	14.37	20.92	Fenugreek, Fennel seeds	Whole Foods	No
Dosa	2.19	2.98	2.52	Black gram (all stores)	Walmart	No
Pulao	10.77	11.34	8.88	Dried onion (all stores)	Walmart	No
Curry	14.26	20.78	24.19	Garam Masala [2], Cardamom [1]	Whole Foods	No
Chai	8.19	8.69	11.43	None	Whole Foods	Yes

## Data Availability

The datasets presented in this article are not readily available because of IRB approval contingency does not allow for release.
